# Hydatid cyst of the clavicle mimicking a bone tumor: a rare case report and literature review

**DOI:** 10.1097/RC9.0000000000001030

**Published:** 2026-09-28

**Authors:** Seyed Zeynab Seyedjavadeyn, Seyed Rouhollah Miri, Fereshteh Ameli, Muhammad Anwar Rahimdeen

**Affiliations:** a Department of General Surgery, Subdivision of Surgical Oncology, Cancer Institute of Iran, Tehran University of Medical Sciences, Tehran, Iran; b Cancer Research Center, Cancer Institute of Iran, Tehran University of Medical Sciences, Tehran, Iran; c Department of Pathology, Cancer Institute of Iran, Imam Khomeini Hospital Complex, Tehran University of Medical Sciences, Tehran, Iran

**Keywords:** bone tumor mimic, clavicle, Echinococcus granulosus, osseous hydatid disease

## Abstract

**Introduction and importance:**

Osseous hydatid disease is a rare manifestation of *Echinococcus granulosus* infection, accounting for 0.5–4% of hydatid disease cases. Clavicular involvement is exceptionally uncommon and may mimic neoplastic or infectious bone lesions, creating diagnostic challenges.

**Case presentation:**

We report the case of a 34-year-old man with a history of a surgically treated left pulmonary hydatid cyst 10 years earlier who presented with progressive swelling over the right clavicle. Imaging revealed a multicystic osteolytic lesion with cortical destruction and soft-tissue extension. The patient underwent preoperative albendazole therapy followed by partial claviculectomy. Histopathological examination confirmed hydatid disease.

**Clinical discussion:**

Osseous hydatid disease can be challenging to diagnose because of its similarity to neoplastic and infectious bone lesions. Complete surgical excision combined with antiparasitic therapy remains the mainstay of treatment to achieve local control and reduce the risk of recurrence.

**Conclusion:**

Hydatid disease should be considered in the differential diagnosis of atypical or destructive clavicular lesions, particularly in patients from endemic regions or with a history of cystic echinococcosis.

## Introduction

Osseous involvement in cystic echinococcosis (CE) is rare, accounting for only 0.5–4% of human hydatid disease (HD) cases^[^1^]^. Bone HD most commonly affects the spine, pelvis, and long bones because of their rich trabecular structure and vascular supply^[^2^]^. Unlike visceral hydatid cysts, osseous lesions lack a well-defined pericyst, allowing progressive infiltration of the medullary cavity and destruction of trabecular bone^[^3^]^.

HIGHLIGHTS

- Clavicular hydatid disease is extremely rare.
- It may mimic bone tumors.
- Imaging findings are nonspecific.
- Surgery is the main treatment.
- Albendazole reduces recurrence.

Clavicular involvement is extremely uncommon. Given the limited cancellous bone within the clavicle and the rarity of disease at this site, clavicular HD may mimic primary bone tumors, metastatic lesions, chronic osteomyelitis, and other cystic bone lesions, resulting in diagnostic challenges^[^3–6^]^.

We present a rare case of clavicular HD in a 34-year-old man with a remote history of pulmonary CE, highlighting the diagnostic and therapeutic considerations of this uncommon presentation.

This work has been reported in line with the SCARE criteria^[^7^]^.

## Case presentation

A 34-year-old man presented with a 2-year history of a gradually enlarging swelling over the right clavicle. There was no history of trauma, constitutional symptoms, or weight loss.

His medical history was significant for a left pulmonary hydatid cyst, which had been surgically excised 10 years earlier. According to the patient, there had been no recurrence; however, previous medical records and histopathology reports were unavailable. He had no history of malignancy, tuberculosis, or other chronic infections.

Physical examination revealed a firm, non-tender, well-defined swelling over the midshaft of the right clavicle. The overlying skin was intact, with a visible scar from the previous biopsy. No lymphadenopathy was detected, and shoulder range of motion and neurovascular examination were normal.

Approximately 4 months before referral to our institution, the patient underwent an open incisional biopsy at another center for a suspected bone lesion. Histopathological examination confirmed HD, and he was referred for definitive management. Chest computed tomography (CT) demonstrated a non-enhancing multicystic lesion measuring 5 × 7 × 3 cm in the mid-shaft of the right clavicle, with cortical irregularity and extension into the supraclavicular soft tissues (Fig. 1A-C). Magnetic resonance imaging (MRI) was not performed because CT adequately defined the extent of bone destruction and soft tissue involvement for surgical planning. Systemic evaluation showed no additional hydatid lesions. Laboratory investigations, including complete blood count and inflammatory markers, were normal, while *Echinococcus granulosus* IgG serology was positive.

**Figure 1.**
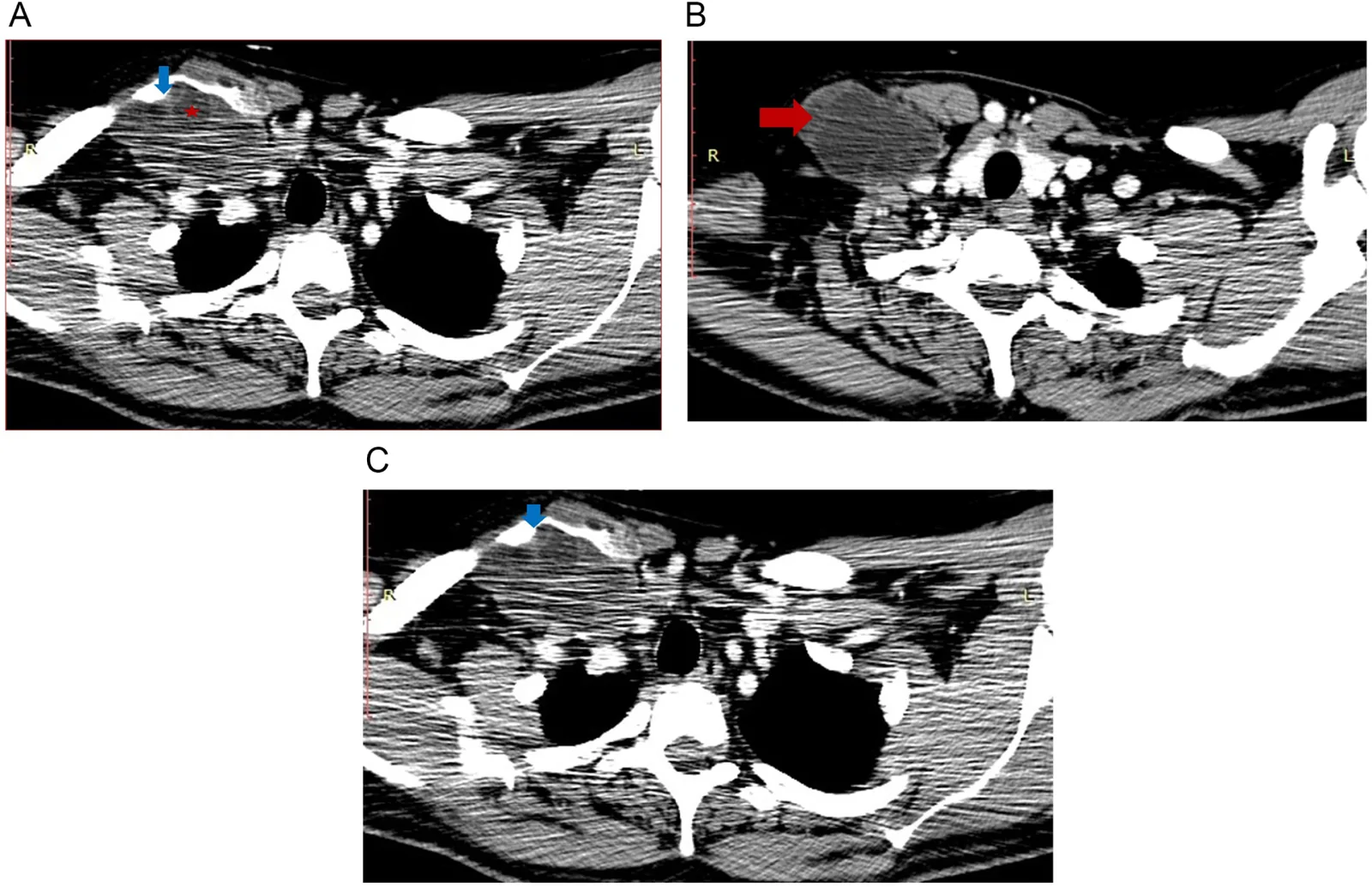
(A) Axial CT image demonstrating a multiloculated osteolytic lesion involving the middle third of the right clavicle (star), showing cortical destruction (arrow) and extension into the adjacent supraclavicular soft tissues. (B) Axial CT image demonstrating the multiloculated cystic lesion (red arrow) involving the middle third of the right clavicle. (C) Axial CT image demonstrating cortical destruction and thinning of the involved clavicle, with cortical irregularity (blue arrow).

Following a multidisciplinary discussion, albendazole (400 mg twice daily) was administered for 3 months before surgery. Partial claviculectomy was subsequently performed. Intraoperatively, a hydatid cyst measuring approximately 7 × 5 cm involved the middle third of the clavicle, with multiple daughter cysts and extensive cortical destruction (Fig. 2A-D). The operative field was carefully protected to minimize the risk of dissemination. Reconstruction or osteosynthesis was not required, as shoulder stability and function were preserved. Hypertonic saline irrigation was used as a scolicidal agent, and no residual cysts were identified in the surrounding soft tissues.

**Figure 2.**
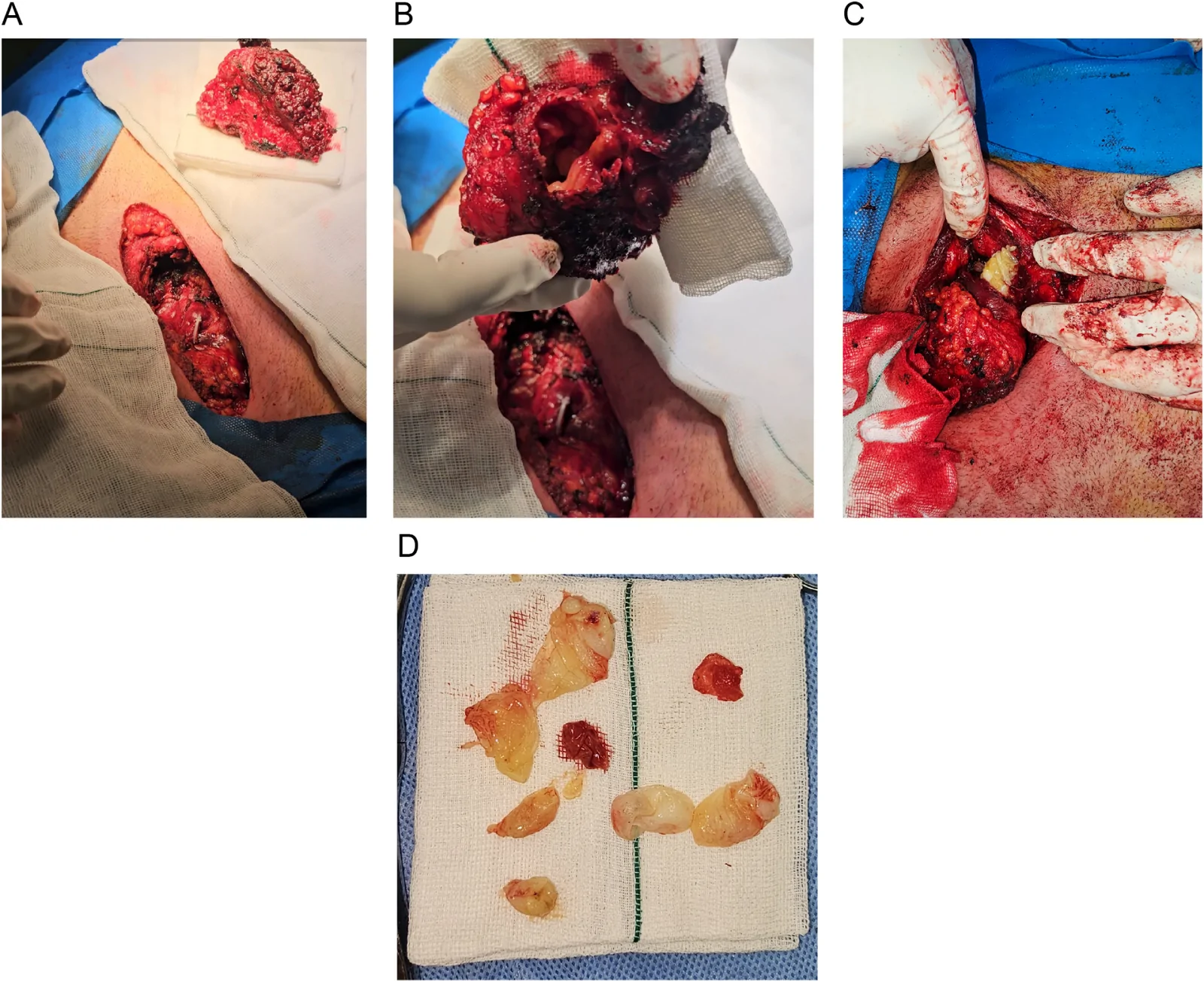
(A) Intraoperative view demonstrating exposure of the clavicle after partial claviculectomy. (B) Resected clavicular specimen demonstrating extensive osseous destruction and multiple daughter cysts. (C) Intraoperative findings demonstrating multiple daughter cysts within the clavicle. (D) Multiple daughter hydatid cysts removed from the clavicular lesion during surgery.

Histopathological examination confirmed HD following complete excision. Microscopic evaluation demonstrated fragments of a laminated, acellular cyst wall composed of thick, eosinophilic, concentrically layered membranes. The laminated membrane was periodic acid–Schiff (PAS)-positive and lacked cellular components, consistent with the ectocyst of *Echinococcus* species. Adjacent host tissue showed a fibrous reaction with focal chronic inflammatory infiltrates, and no evidence of malignancy was identified (Fig. 3A-B).

**Figure 3.**
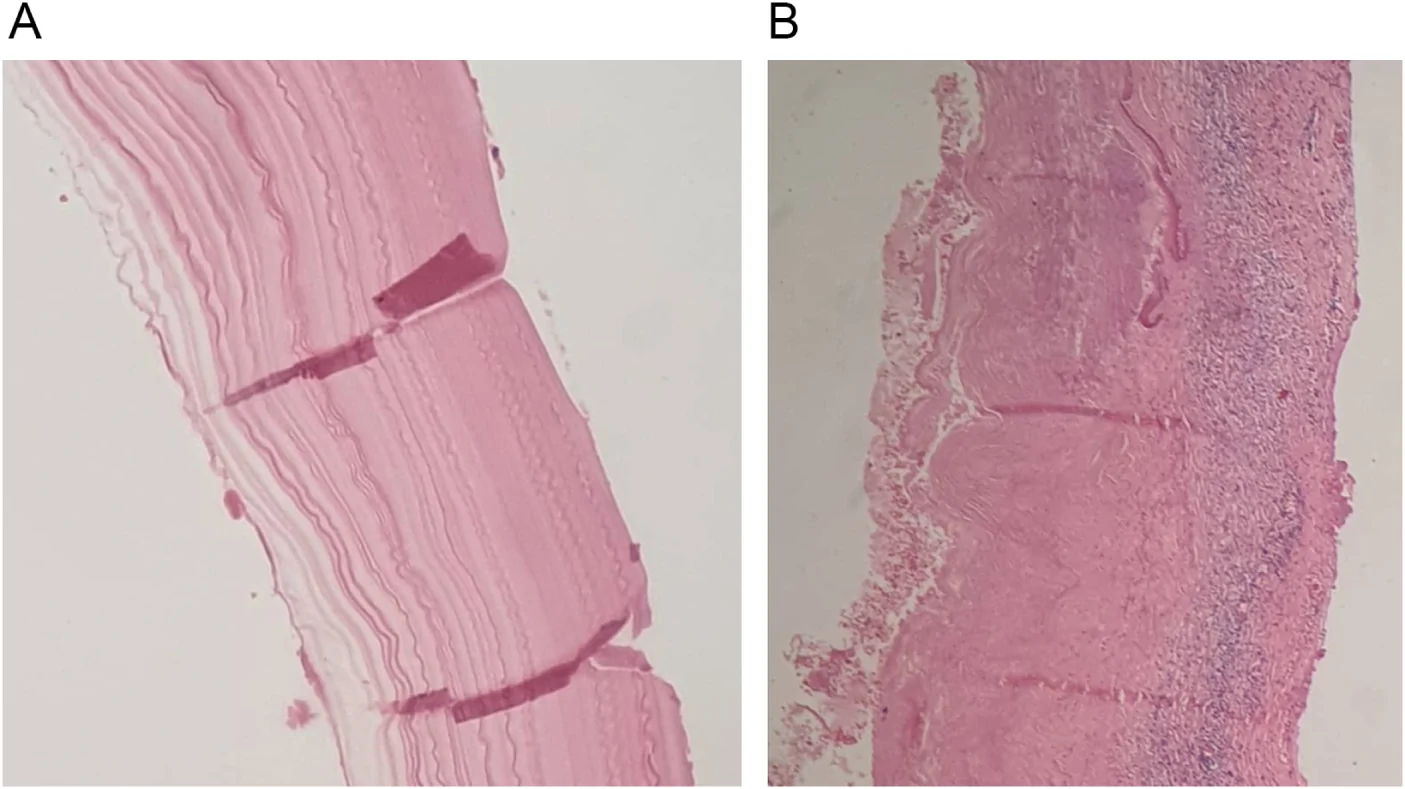
(A) Histopathological examination demonstrating a laminated, acellular cyst wall characteristic of hydatid disease (PAS-positive laminated membrane). (B) Higher-power photomicrograph demonstrating the PAS-positive, laminated, acellular membrane characteristic of hydatid disease.

The postoperative course was uneventful. Shoulder range of motion was preserved, and cosmetic deformity was minimal. At the 12-month follow-up, the patient remained asymptomatic, with preserved shoulder function and no clinical or radiological evidence of recurrence.

## Discussion

Hydatid disease (HD) is a zoonotic infection with a worldwide distribution, particularly affecting endemic pastoral regions^[^8^]^. Although it most commonly involves the liver and lungs, osseous HD accounts for only 0.5–4% of cases and represents a challenging manifestation due to its infiltrative growth and nonspecific clinical and radiological features^[^1–3^]^. Unlike visceral cysts, bone lesions lack a well-defined pericyst, allowing daughter cysts to spread through the medullary cavity and cause progressive trabecular destruction, cortical erosion, and soft-tissue extension. Its indolent course and nonspecific radiological findings often delay diagnosis for years after disease onset^[^3,9^]^.

Bone hydatid cysts typically appear as multiloculated, expansile osteolytic lesions on CT and radiographs, with variable degrees of cortical destruction. MRI is particularly valuable for demonstrating daughter cysts and evaluating soft tissue extension. The absence of contrast enhancement may provide an important clue in differentiating hydatid lesions from malignant and infectious bone lesions. Differential diagnoses include abscess, tuberculosis, metastatic disease, and primary bone tumors^[^10^]^. In our case, the combination of a multiloculated osteolytic lesion, cortical destruction, and soft tissue extension initially suggested a primary bone tumor; however, the patient’s history of pulmonary HD and the imaging characteristics supported the diagnosis of osseous HD.

The most frequently affected skeletal sites in osseous echinococcosis include the spine, pelvis, femur, and tibia, where abundant cancellous bone and vascularity may facilitate parasite dissemination. Clavicular involvement is exceedingly rare, possibly due to the limited cancellous bone and vascular supply of the clavicle compared with more commonly affected sites^[^1–3,11^]^.

To the best of our knowledge, only two well-documented cases of clavicular HD have been reported. Takakuwa *et al* reported proximal clavicular involvement, successfully treated with surgical excision and long-term antiparasitic therapy, with no recurrence during follow-up^[^4^]^. Wang *et al* (2023) described a case with extensive clavicular destruction and soft-tissue extension, managed by total claviculectomy, resulting in preserved shoulder function and no recurrence at 1 year^[^5^]^. Similar to these reports, our patient achieved a favorable outcome with surgical excision and adjuvant albendazole therapy. However, unlike Wang *et al*, partial claviculectomy was performed in our case because the disease was limited to the middle third of the clavicle, allowing complete resection while preserving shoulder function.

To better contextualize our findings, a comparison of previously published cases of clavicular HD with the present case is presented in Table 1.

**Table 1:** Comparison of previously reported cases of clavicular hydatid disease with the present case.

Author (year)	Age/sex	Clavicle involvement	Previous hydatid disease	Surgical treatment	Adjuvant albemdazole	Follow-up	Outcome
Takakuwa *et al* (2002)^[^4^]^	47/Female	Proximal clavicle	Not reported	Partial claviculectomy	Yes	9 years	No recurrence
Wang *et al* (2023)^[^5^]^	29/Female	Extensive clavicular involvement	No	Total claviculectomy	Yes	12 months	No recurrence
Present case	34/Male	Middle third of the right clavicle	Yes; Previous pulmonary hydatid cyst	Partial claviculectomy	Yes	12 months	No recurrence

The treatment of HD may be medical, surgical, or a combination of both. In osseous HD, complete surgical excision remains the cornerstone of treatment, although the extent of resection depends on the anatomical involvement. Accordingly, partial or total claviculectomy may be required for clavicular lesions. In our case, the lesion was confined to the middle third of the clavicle without involvement of the sternoclavicular or acromioclavicular joints, allowing complete resection of the affected segment while preserving shoulder stability and function. Reconstruction or osteosynthesis was therefore not required. Adjuvant albendazole therapy may be beneficial, particularly in cases of incomplete resection or a high risk of recurrence; however, its efficacy as monotherapy is limited because of poor penetration into bone tissue^[^1,11^]^. In our case, combined surgical and medical therapy resulted in a favorable outcome without recurrence during follow-up.

A major diagnostic challenge in osseous HD is its resemblance to primary bone tumors, metastatic lesions, and chronic osteomyelitis. Although biopsy may aid in diagnosis, it carries a potential risk of cyst rupture and dissemination. In our patient, an incisional biopsy performed before referral confirmed the diagnosis despite the potential risk associated with this approach.

Overall, the diagnosis of osseous HD requires a high index of suspicion, particularly when destructive bone lesions occur in patients with a history of HD. Integration of clinical history, imaging findings, and histopathological examination is essential for accurate diagnosis and appropriate management.

## Conclusion

HD should be considered in the differential diagnosis of atypical or destructive clavicular lesions, particularly in patients from endemic regions or with a history of CE. Accurate diagnosis requires integration of clinical history, imaging findings, and histopathological examination. Surgical excision combined with appropriate antiparasitic therapy may achieve favorable functional outcomes.

## Data Availability

Data supporting this study are available from the corresponding author upon reasonable request.
